# Ultrafast Cation–Dication Dynamics in Ammonia Borane: H‑Migration to Roaming H_2_ and Reduced H_3_^+^ Formation under Strong-Field Ionization

**DOI:** 10.1021/acs.jpca.5c07228

**Published:** 2026-02-20

**Authors:** Sung Kwon, Naga Krishnakanth Katturi, Bruno I. Moreno, Carlos Cárdenas, Marcos Dantus

**Affiliations:** † Department of Chemistry, 3078 Michigan State University, East Lansing, Michigan 48824, United States; ‡ Department of Physics, CEDENNA, University of Chile, Las Palmeras, 3425, Ñuñoa 7800003, Chile; § Department of Physics and Astronomy, 3078 Michigan State University, East Lansing, Michigan 48824, United States; ∥ Department of Electric and Computer Engineering, 3078 Michigan State University, East Lansing, Michigan 48824, United States

## Abstract

We report a femtosecond time-resolved strong-field study of ammonia borane (AB, BH_3_NH_3_) following both single and double ionization, revealing ultrafast fragmentation dynamics and hydrogen release. Time-resolved mass spectrometry and ab initio molecular dynamics simulations are used to identify the molecular origin of the neutral and ionic products. Singly ionized AB produces neutral H and H_2_, while doubly ionized AB produces neutral H and H_2_ along with H^+^, H_2_^+^, and H_3_^+^, all within 1 ps. Electronic-structure calculations show that H, H^+^, H_2_, H_2_^+^, and H_3_^+^ originate predominantly from hydrogen atoms bound to the boron center and that their formation proceeds through hydrogen migration and, in some channels, neutral H_2_ roaming. The calculations further indicate that the dication meets the structural and energetic requirements for neutral H_2_ release, a prerequisite for forming astrochemically relevant H_3_^+^. However, the large adiabatic relaxation energy causes most roaming H_2_ to dissociate before proton abstraction, suppressing H_3_^+^ formation. These results provide new insight into dissociative ionization pathways in hydrogen-rich molecules, extend mechanistic principles developed for halogenated alkanes to ammonia borane, and suggest implications for hydrogen-release chemistry in ammonia-borane-based storage materials.

## Graphical abstract

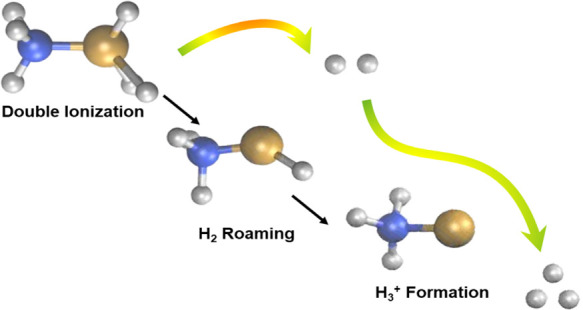

## Introduction

Ammonia borane (AB, BH_3_NH_3_) has attracted significant attention as a chemical hydrogen storage material due to its exceptionally high hydrogen content, 19.6 wt %, which exceeds that of many conventional storage materials.^1–4^ The dehydrogenation process of AB is a key area of research, given its relevance to hydrogen storage applications. Experimental studies have shown that thermal decomposition releases hydrogen in a stepwise manner, forming intermediates such as polyaminoborane and borazine.^5^ While most studies have focused on condensed-phase or thermal dehydrogenation behavior of AB, little is known about its gas-phase dynamics under ionizing conditions. The electron-ionization mass spectrum of AB is not available in existing databases, and little is known about its fragmentation behavior upon ionization. In this study, we examine the ultrafast, time-resolved dissociative dynamics of AB following strong-field double ionization.

The chemical bonding in AB involves a dative (coordinate covalent) bond, in which the nitrogen atom donates a lone pair of electrons to the electron-deficient boron atom. The structural properties of AB have been extensively studied to understand its hydrogen storage capabilities. Photoemission and X-ray absorption studies have been conducted to probe its valence and core electronic states, shedding light on the nature of bonding and electronic transitions within the molecule.^6^ Theoretical investigations using ab initio molecular dynamics and metadynamics have provided insights into the initial stages of dehydrogenation, suggesting the formation of intermediates like ammonia diborane, which can lead to autocatalytic hydrogen production cycles.^7^ Recent studies have also explored the potential of ammonia borane in carbon dioxide capture and conversion.^8,9^

Several molecules have been shown to release H_3_^+^ upon double ionization. For example, Eland demonstrated that H_3_^+^ can be produced from a range of organic molecules through collisions with high-energy electrons or excitation by 30.4 nm photons.^10^ Similarly, Yamanouchi and co-workers reported H_3_^+^ ejection from gas-phase methanol under intense 800 nm laser fields.^11,12^ Complementing these studies, our group has been investigating ultrafast, far-from-equilibrium chemical processes initiated by interactions between molecules and secondary electrons.^13,14^ In particular, we have focused on the formation dynamics and mechanisms of H_3_^+^ generation from various organic molecules following strong-field ionization.^15–20^ Using EUV excitation, Strasser and collaborators have also investigated H_3_^+^ formation dynamics in several of these same systems, including methanol and ethanol.^21,22^ Despite the prevalence of organic precursors in these studies, only a few nonorganic molecules, such as AB examined here, and GeH_4_, have been shown to generate H_3_^+^ upon ionization.^10^ The production of H_3_^+^ is significant beyond laboratory conditions, it is a key ion in interstellar chemistry, acting as a catalytic proton donor,^23,24^ and has recently been proposed as an indirect probe for dark matter.^25^ Interestingly, AB is isoelectronic with ethane, a molecule known to readily form H_3_^+^ via strong-field dissociation.^26–30^ This structural similarity raises questions about the potential of AB to follow analogous dissociation pathways in the gas phase.

## Methods

The ultrafast disruptive-probing method used to monitor all reaction pathways following strong-field ionization has been detailed previously.^31^ In this case, a Ti:sapphire laser centered at 795 nm, delivering 65 fs pulses at 1 kHz as measured by in situ autocorrelation, served as the ionization source. Ammonia borane (95% purity, Sigma-Aldrich) was used without further purification. Laser pulses were focused into a Wiley–McLaren time-of-flight (TOF) mass spectrometer using a 200 mm lens, with polarization aligned parallel to the TOF axis. Each pulse was split into a strong pump pulse to ionize the molecule and a weak probe pulse to disrupt product formation. Laser intensities were calibrated by measuring the Ar^2+^/Ar^+^ ion ratio.^32^ Ammonia borane vapor was introduced into the TOF chamber as an effusive beam after a freeze–pump–thaw cycle. Room-temperature sublimation provided sufficient vapor to obtain the experimental data. During data acquisition, the chamber pressure was held at 1 × 10^–5^ Torr. The baseline vacuum pressure was 9 × 10^–8^ Torr and returned to this level within a few seconds of closing the valve. Ion signals were digitized with a 1 GHz oscilloscope (LeCroy WaveRunner 610Zi). The TOF was set to detect cations, so only positively charged ions appear in the mass spectra. To account for the natural isotopic distribution of boron (80% ^11^B and 20% ^10^B), peaks containing boron, except the molecular ion, were corrected by 20% to minimize isotope-related contributions. Additionally, contributions from air in the spectrum were removed, using the ratio of ionized N_2_^+^ to O_2_^+^ in air under identical laser conditions to subtract the N_2_^+^ contribution from the *m*/*q* 28 signal.

A total of *N* = 39,660 single-shot mass spectra of AB were acquired and analyzed for the kinetic energy release (KER) distributions for H^+^, H_2_^+^, and H_3_^+^.

Electronic structure calculations, optimization of the ground state and dication geometries of AB, adiabatic relaxation energies, and H_2_ dissociation energies were carried out at the CCSD­(T)^33–36^ /aug-cc-pVDZ^37,38^ level of theory using GAMESS 2019.R1.^39^ The ionization potential calculations for the monocation were done at the CCSD^35,36,40,41^ /cc-pVTZ^37,42^ and dication were done at the DIP-EOMCC­(4h-2p)^43–47^ /cc-pVTZ^37,42^ level of theory using the same version of GAMESS. All molecular dynamics simulations were performed using Gaussian 09 ^48^ evaluating the force with DFT using the ω-B97XD^49^ exchange–correlation functional with a 6-311+G­(d,p) basis set. To better capture the potential energy of bond breaking processes, a spin broken-symmetry solution was allowed for trajectories. Equations of motion were integrated with Verlet-velocity and a Hessian-based integrator^50^ with step size of 0.5 fs and $0.25\;\sqrt{amu}\cdot bohr$ , respectively. For the cation’s AIMD trajectories, atomic positions were taken from a molecular dynamics (MD) simulation of the neutral AB in the *NV* *E*(conserving the number of particles, volume, and kinetic energy) ensemble at 300 K (with 0 eV of additional energy). The velocities were sampled from a Maxwell–Boltzmann distribution (thermal sampling), with the constraint that the total kinetic energy of the nuclei was scaled to 1, 1.5, or 7.8 eV. For the case labeled as 0 eV, the velocities were obtained directly from the *NVE* MD simulation (Maxwell–Boltzmann at 300 K). A total of 100 trajectories were generated for each energy level.

For the dication’s AIMD trajectories, two distinct cases were considered. In the first case, a set of 200 trajectories was generated, where the positions and velocities were derived from *NVE* molecular dynamics simulations of the neutral state (Maxwell–Boltzmann distribution at 300 K). In this scenario, the adiabatic energy is sufficient to promote hydrogen (H) release but not enough to induce Coulomb fragmentation of the boron–nitrogen (B–N) bond. To observe B–N bond fragmentation (second case), 4 eV of energy was selectively injected into the normal mode of BH_3_NH_3_^2+^ with the largest B–N stretching character, using quasi-classical sampling. This approach was chosen because B–N bond cleavage is a rare event compared to H release, requiring thousands of thermally sampled trajectories to observe it. In some trajectories, fragmentation did not lead to bond breaking but rather to additional hydrogen release, producing BHNH_3_^2+^. For these cases, we further injected 4 eV into the B–N stretching mode of BHNH_3_^2+^, and so on. Only 25 trajectories were run for these higher-energy cases, as increasing the number of trajectories did not significantly affect the observed trends.

## Results and Discussion

The experimental high-intensity selected strong-field ionization mass spectrum of BH_3_NH_3_ resulting from the difference between two different laser peak intensities of 3.1 × 10^14^ W/cm^2^ and 2.7 × 10^14^ W/cm^2^ is shown in Figure 1a. This difference intensity minimizes contributions from the lower-intensity regions of the Gaussian focal-volume intensity distribution.^51–53^ The mass spectrum shows that under strong-field ionization AB can lose all its hydrogen atoms. In addition, dication species are observed at *m*/*q* 15.5 (M^2+^), 14.5 (M – 2H)^2+^, 13.5 (M – 4H)^2+^, and 5.5 (B^2+^). All smaller fragment ions, beginning with NH_3_^+^, exhibit Coulomb-explosion signatures in the time-of-flight spectrum, characterized by distinct forward and backward peaks, except for those at *m*/*q* 15.5, 15, 14.5, and 13.5, which appear as single peaks and are assigned as the molecular dication or the molecular dication with the loss of one to three hydrogen atoms. We note that the *m*/*q* 15 channel may also contain a contribution from NH^+^. The low-intensity 1.3 × 10^14^ W/cm^2^ ionization mass spectrum is shown in Figure 1b. This spectrum shows that even at low intensities, AB loses one or more H atoms. The high yield of *m*/*q* 30, corresponding to loss of a single hydrogen atom, is attributed to the small energy gap between the adiabatic ionization potential of AB (9.6 eV) and the threshold for the first H atom loss (10.0 eV).^54^ Hydrogen loss is quantified in Tables 1 and 2 from the integrated peak areas for the monocation and dication, respectively, and both tables show that hydrogen loss is frequent following both single and double ionization.

**Figure 1:**
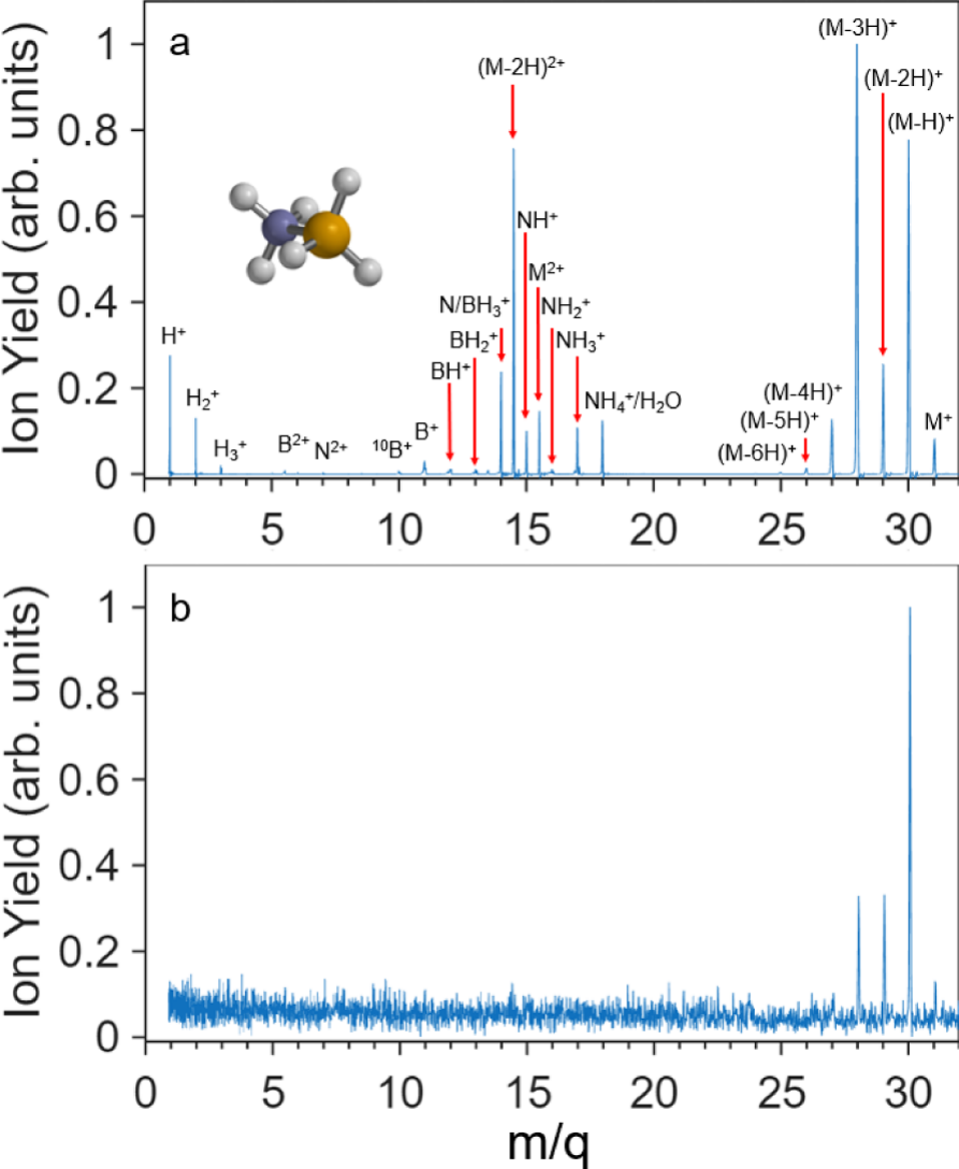
(a) Strong-field ionization mass spectrum of ammonia borane after the difference between two different laser peak intensities of 3.1 × 10^14^ W/cm^2^ and 2.7 × 10^14^ W/cm^2^. Fragment peaks are labeled with their sum formulas. (b) Low-intensity strong-field ionization mass spectrum of ammonia borane taken at an intensity of 1.3 × 10^14^ W/cm^2^. Both spectra are normalized to a maximum intensity of 1. M corresponds to the molecular ion.

**Table 1:** Experimental Integrated Areas of *m*/*q* Fragments Corresponding to H Loss Normalized by the Sum of the Total Integrated Area of All Peaks at an Intensity of 1.3 × 10^14^ W/cm^2^

*m*/*q*	area (% of monocations)
31	7.5
30	38.6
29	13.9
28	32.8
27	7.3

**Table 2:** Experimental Areas of *m*/*q* Fragments Corresponding to Loss of 2H(s) Normalized to the Total Area of Dication Peaks at an Intensity of 3.1 × 10^14^ W/cm^2^

*m*/*q*	area (% of dications)
15.5	15.3
14.5	80.2
13.5	1.4

Dissociative ionization was further examined through calibrated^32^ laser power dependence in Figure 2, where each successive data point corresponds to a higher intensity. For each fragment, we compute the numerical difference in integrated yield between adjacent points (i.e., point *n* + 1 minus point *n*), which suppresses the focal-volume contribution. The resulting difference trace is then fit with an error function. The appearance of each fragment was determined by the laser peak intensity at which its yield exceeded 10% of the maximum (see Figure S1).

**Figure 2:**
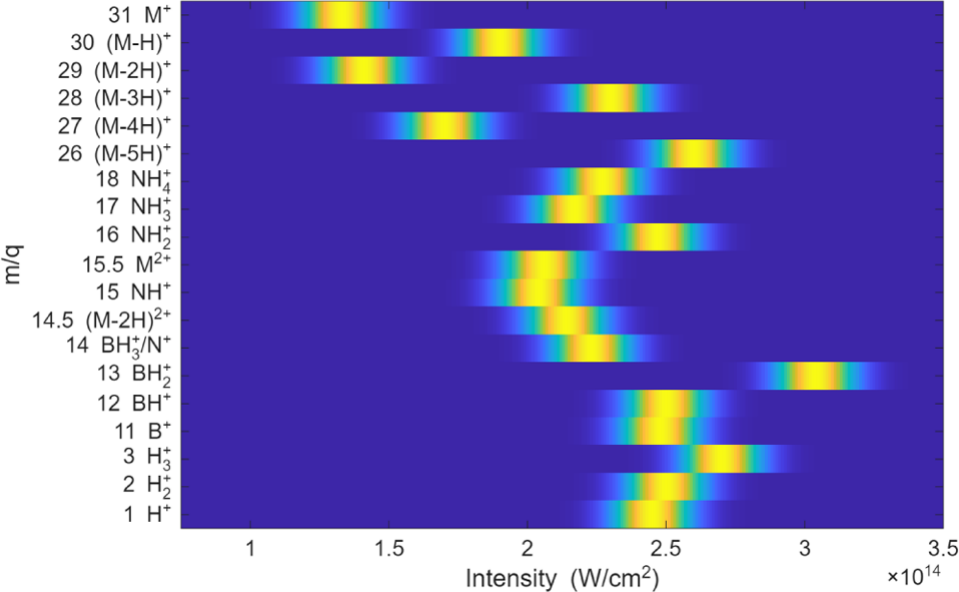
Heat map of the major AB fragment yields as a function of calibrated laser intensity. The appearance-intensity thresholds, which are proportional to the corresponding appearance energies, were extracted using the procedure described in the Supporting Information (Figure S1). The width of each onset feature reflects the uncertainty in the threshold determination and thus provides a measure of confidence.

When photoionization occurs within a single optical cycle or faster, the liberated electron gains kinetic energy proportional to the peak intensity. The ponderomotive energy, which scales linearly with intensity, governs the electron’s motion, driving it away from the ion and back toward it as the laser field reverses.^55^ In general, the intensity required for ion formation can be directly related to the fragment’s appearance energy (AE). Upon recollision, the electron transfers energy to the molecule, inducing ionization, fragmentation, and in some cases, double ionization. The calculated single and double vertical ionization potentials for AB were calculated to be 11.9 and 31.1 eV, see below at the CCSD^40,41^ /cc-pVTZ^37,42^ and DIP-EOMCC­(4h-2p)^43–47^ /cc-pVTZ^37,42^ respectively. In general, we see that losing pairs of hydrogen atoms requires less energy compared to single or triple hydrogen loss. Unlike most hydrocarbons,^56^ H^+^ loss from AB is observed only when the laser intensity is high enough to cause double ionization. Note that fragments resulting from breaking the B–N bond (*m*/*q* 18, 17, 16, 14, 13, 12, and 11) exceed the appearance energy of the dication.

The ultrafast formation dynamics of all ionic species were simultaneously measured using disruptive probing, a technique where an intense pump pulse ionizes the molecule and a time-delayed weak probe pulse disrupts its fragmentation.^31^ The probe pulse by itself causes no ionization. The resulting ion yields as a function of pump–probe delay time enable the simultaneous tracking of all fragmentation pathways and products.^31^ Figure 3 shows the ion yield as a function of pump–probe delay for all major fragments of AB.

**Figure 3:**
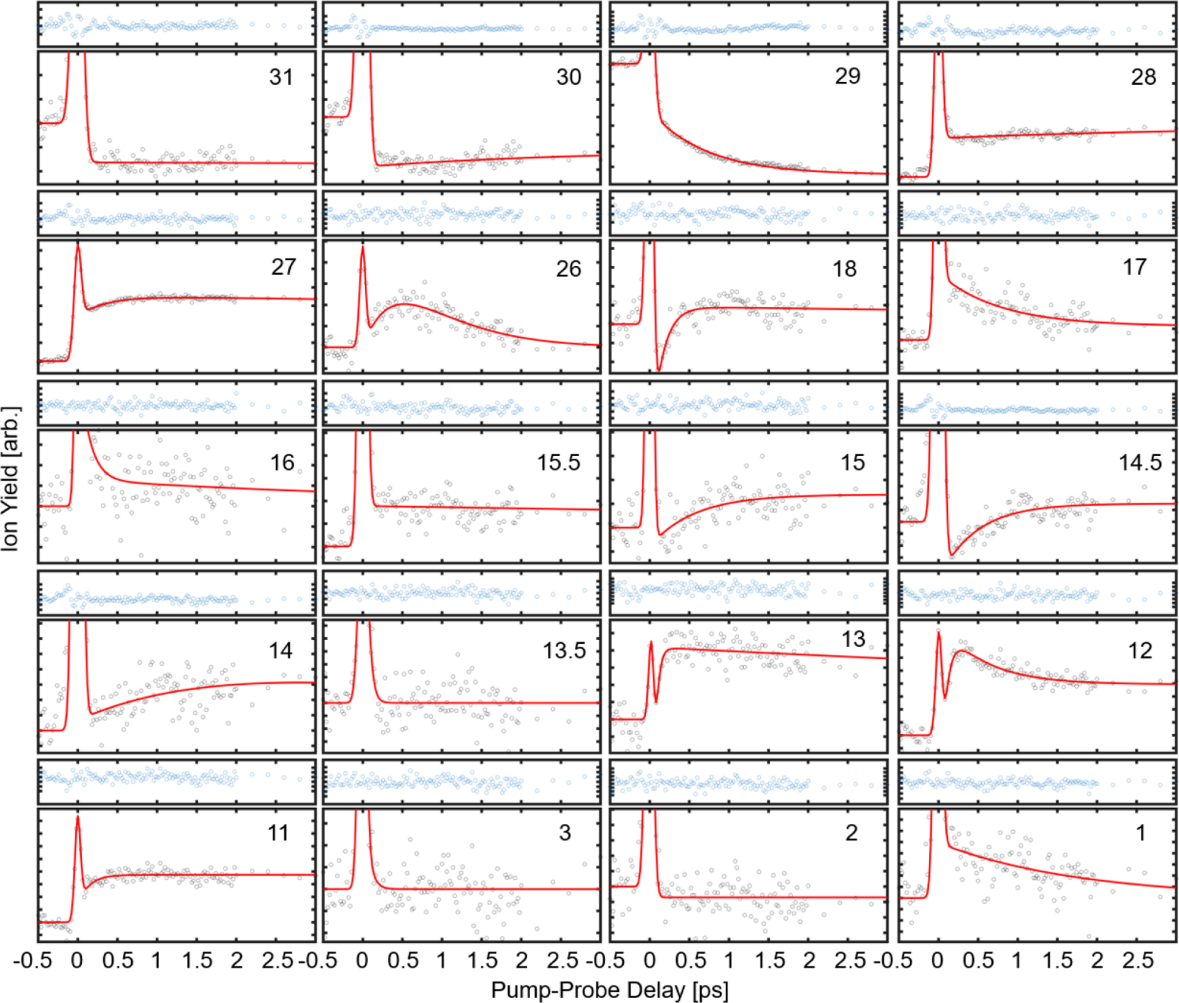
Ion yield as a function of pump–probe delay for all major *m*/*q* with the residual plotted above. The time-resolved data was acquired at a pump intensity of 3.1 × 10^14^ W/cm^2^ and probe intensity of 4.4 × 10^13^ W/cm^2^.

The time-dependent yield of the selected fragments exhibit a narrow feature at zero time delay, corresponding to the spatial and temporal overlap of the pump and probe pulses, as shown in Figure 3. At asymptotic pump–probe delays, the ion yields for *m*/*q* 31, 30, and 29 remain reduced relative to their negative-delay baselines. In contrast, the yields of all other major fragments either (i) recover to the negative-delay value—indicating that, at long delays, the probe no longer measurably perturbs their formation or depletion—or (ii) increase above the baseline, consistent with probe-induced formation from a long-lived precursor. At early (subpicosecond) delays, transient depletions or enhancements reflect probe perturbation of the evolving reaction dynamics; fitting these transients provides an effective formation time scale for the corresponding product. Owing to the low signal-to-noise ratio, we could not reliably extract a formation time scale for H_3_^+^. Loss of two hydrogens leading to *m*/*q* 29 displays a depletion with a time constant of 340 ± 65 fs and a long offset term, while loss of four hydrogens leading to *m*/*q* 27 shows a fast rise with a 340 ± 100 fs time constant and a long offset term. The similarity between these time scales suggests that both two- and four-hydrogen loss channels proceed through closely related dynamics. On the other hand, the loss of five hydrogen atoms leading to *m*/*q* 26 shows a rapid enhancement with a 175 ± 10 fs time constant, followed by a slower decay with a 810 ± 35 fs time constant. A similar enhancement is observed for *m*/*q* 13 and 12, though it occurs more rapidly, with time constants of 40 ± 40 fs and 80 ± 44 fs, respectively. The formation of NH_4_^+^ (*m*/*q* 18) is characterized by a time constant of 140 ± 47 fs. This rapid hydrogen migrations from boron to nitrogen occurs on a time scale consistent with previously reported H-transfer dynamics.^57–59^ All the fitting constants are provided in Table 3. In certain cases (*m*/*q* = 29, 27, and 26), the dynamics were corrected for contamination from ^10^B. This correction, applied from high to low *m*/*q*, was based on isotope contributions estimated from the ^11^B-corrected fragment signal. Specifically, the ion yield of the affected *m*/*q* was scaled at each time delay using its corresponding correction factor. For *m*/*q* 31, 30, 28, and 26, isotope contributions were less than 3% and thus not corrected. The dynamics observed under high-intensity conditions closely match those at low intensity (see Figure S2), indicating that fragments in the *m*/*q* 26–31 range predominantly arise from monocationic AB, even at high intensity. The dication corresponding to (M – 2H)^2+^ exhibits a depletion with a time constant of 480 ± 55 fs, consistent with H_2_ loss. Dicationic fragments at *m*/*q* 15.5 and 13.5 show no discernible time-resolved dynamics.

**Table 3:** Exponential Fit Parameters for All *m*/*q* Ion Dynamics from AB

*m*/*q*	τ_1_ (fs)	τ_2_ (fs)	τ_3_ (fs)	*a*_1_	*a*_2_	*a*_3_
31	92 ± 10	offset	-	0.01	–0.004	-
30	250 ± 180	10,000 ± 530	-	–0.002	0.0008	-
29	340 ± 65	offset	-	0.01	–0.01	-
28	3500 ± 1300	offset	-	–0.004	0.01	-
27	340 ± 100	offset	-	–0.001	0.004	-
26	175 ± 10	810 ± 35	-	0.02	–0.03	-
18	140 ± 47	offset	-	–0.02	0.002	-
17	720 ± 75	offset	-	0.005	0.003	-
16	130 ± 20	5100 ± 2300	-	0.01	0.003	-
15.5	offset	-	-	0.004	-	-
15	640 ± 78	offset	-	–0.005	0.003	-
14.5	480 ± 55	offset	-	–0.006	0.002	-
14	660 ± 120	offset	-	–0.003	0.003	-
13.5	40 ± 32	-	-	0.4	-	-
13	40 ± 40	offset	-	–0.1	0.02	-
12	80 ± 44	480 ± 270	offset	–0.07	0.02	–0.02
11	120 ± 16	offset	-	0.02	–0.01	-
3	offset	-	-	0.004	-	-
2	offset	-	-	–0.002	-	-
1	400 ± 200	-	-	0.005	-	-

The time-of-flight arrival of H^+^, H_2_^+^, and H_3_^+^, can be further analyzed to determine their kinetic energy release (KER). In our experiments, the laser polarization was oriented parallel to the time-of-flight (TOF) axis. Because ammonia borane has a strong permanent dipole moment, molecules with their dipole aligned along the laser polarization are expected to ionize most efficiently. As a result, we anticipate strongly polarized forward–backward ejection of the H_*n*_^+^ fragments along the polarization (TOF) axis.

The fragment-ion kinetic energy, *E*_ion_, was obtained from the time separation between the forward and backward peaks due to kinetic-energy release along the TOF axis, Δ*t*, according to

1 $$E_{ion}=\frac{q^{2}F^{2}{\left(\Delta t\right)}^{2}}{8m}$$

where *q* is the fragment charge, *F* is the static extraction field, Δ*t* is the forward–backward flight-time difference, and *m* is the fragment mass. To convert this value to the total kinetic energy release (KER) of the two-body breakup, we account for recoil of the complementary fragment with mass *M* – *m*, where *M* is the parent-ion mass, using

2 $$E_{total}=E_{ion}\left(1+\frac{m}{M-m}\right)$$

These expressions were used consistently for all reported KER values. As shown in Figure 4, the KER distributions for H^+^, H_2_^+^, and H_3_^+^ exhibit prominent peaks at 5.7, 6.1, and 5.8 eV, respectively. These values are consistent with Coulomb explosion following double ionization and are comparable to the KER expected for doubly ionized methanol (5.0 eV if all three H atoms originate from carbon and 5.48 eV if one H atom originates from oxygen).^15^ Because KER is highly sensitive to the interatomic distance at the moment of charge separation, even small changes in geometry can shift the peak substantially; for example, the KER for N_2_ is 6.5 eV.^60^ In addition to the low-KER peaks, H_2_^+^ and H^+^ show higher-energy peaks at 13 and 18 eV, respectively, consistent with fragmentation from more highly charged precursors. Similar high-KER channels have been reported for trications, including triply ionized ethylene at 8 × 10^14^ W/cm^2^, which produces a peak near 11 eV,^61^ and triply ionized acetylene, for which the total KER is 15 eV.^62^

**Figure 4:**
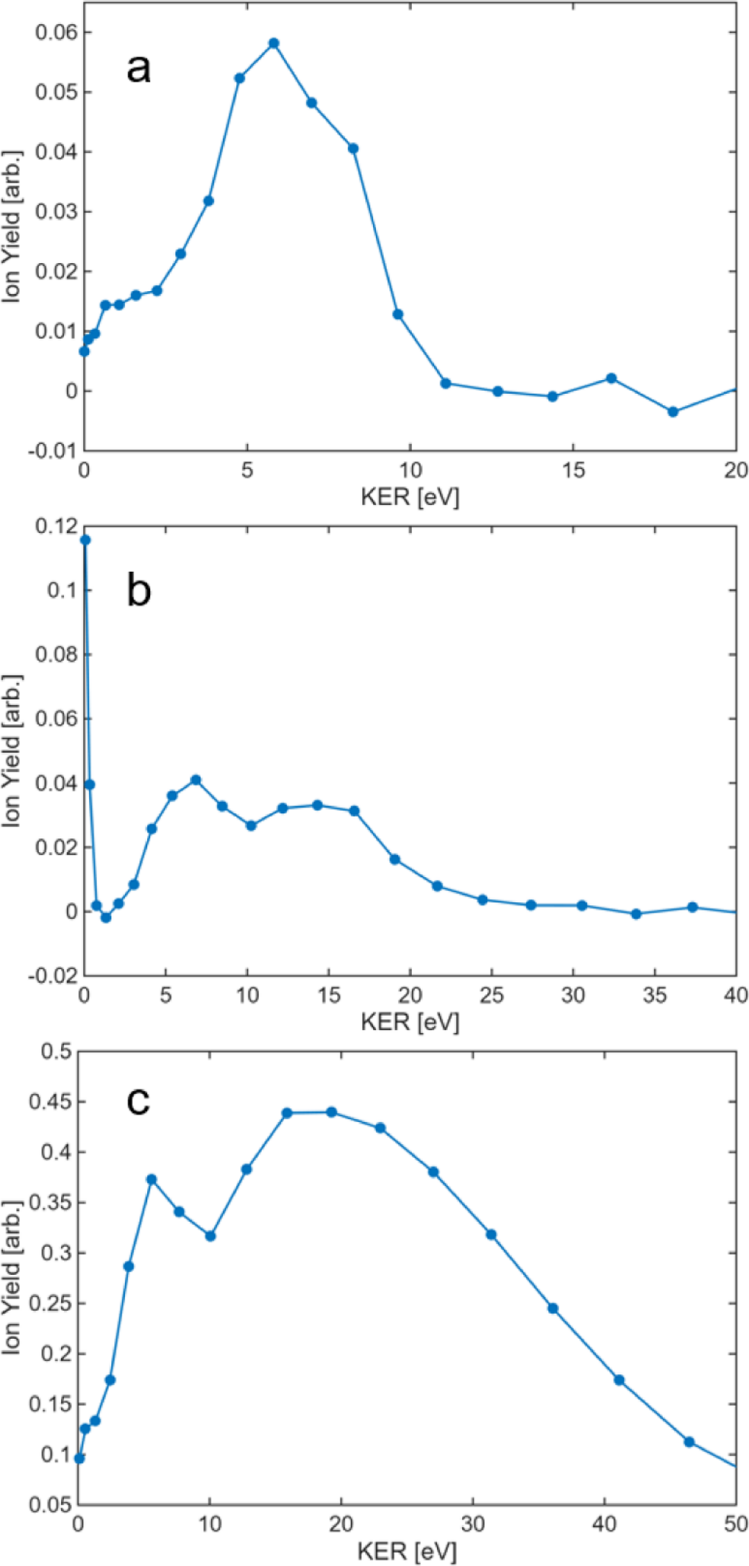
Experimental KER spectra of (a) H_3_^+^, (b) H_2_^+^, and (c) H^+^. Blue dots represent the measured data, while the blue line serves as a guide to the eye.

Using the Coulomb repulsion expression inverted to estimate the charge-separation distance at explosion, we extract mean fragment separations of 2.5, 2.1, and 2.24 Å for the doubly ionized channels producing H^+^, H_2_^+^, and H_3_^+^, respectively, and 1.7 and 2.0 Å for the higher-energy channels assigned to trication precursors producing H and H_2_, respectively. Because the pulses used here are relatively long, bond stretching can occur during the ionization sequence, which favors enhanced ionization at extended bond lengths.^63^ The observation of H^+^ kinetic energy release extending beyond 40 eV is consistent with fragmentation from a highly charged precursor accessed through sequential ionization via enhanced ionization, potentially promoted by proton migration and the associated transient charge localization. If enhanced ionization in this system is promoted by bond stretching, then employing sub-10 fs pulses that are too short for appreciable stretching should curtail the enhanced-ionization pathway and decrease the yield of very high charge states. We plan to test this expectation directly using our recently commissioned 5 fs laser pulses.

Finally, only H_2_^+^ exhibits a near-zero-KER peak. Although a zero-KER component could suggest formation from the monocation, the laser-intensity dependence in Figure 2 indicates that H_2_^+^ appears predominantly at higher intensities and therefore correlates with dication formation. We thus attribute the zero-KER H_2_^+^ signal to ionization of neutral H_2_ (ionization energy 15.4 eV) after it dissociates from the monocation within the same 65 fs laser pulse, before the departing fragments acquire significant relative kinetic energy.

Previous work has shown that the production of H_3_^+^ from methyl pseudohalogens and halogens (CH_3_X) via the H_2_ roaming mechanism^15,16^ is governed by three key factors.^20^ These are summarized below:

1. Double ionization leads to a significant elongation of two C–H bonds compared to the neutral CH_3_X molecule.
2. In the lowest singlet state of CH_3_X^2+^, the distance between the two hydrogen atoms with the elongated bonds should approximate the ground state H_2_ bond length.
3. The adiabatic relaxation energy, defined as the energy difference between the Franck–Condon point (vertical ionization) and the lowest singlet state of the dication, must exceed the dissociation energy of neutral H_2_ for its release, a key step in the roaming pathway, but it cannot be so large that other reactive pathways or decomposition mechanisms compete significantly.

Although AB is not a CH_3_X compound, these governing principles may extend to other molecules, such as ethane,^20^ which upon double ionization undergoes H atom migration and the CH_2_CH_4_^2+^ species releases H_2_ which roams and abstracts a proton to form H_3_^+^.^30^ To determine if AB, which is isoelectronic with ethane, fulfills these requirements, electronic structure calculations were carried out using GAMESS 2019.R1 ^39^ at the CCSD­(T)^35^/aug-cc-pVDZ^37,38^ level of theory. Comparing the ground state and dication geometries of AB, shown in Figure 5, one notices the significantly elongated B–H bonds relative to neutral AB, thus satisfying the first criterion.

**Figure 5:**
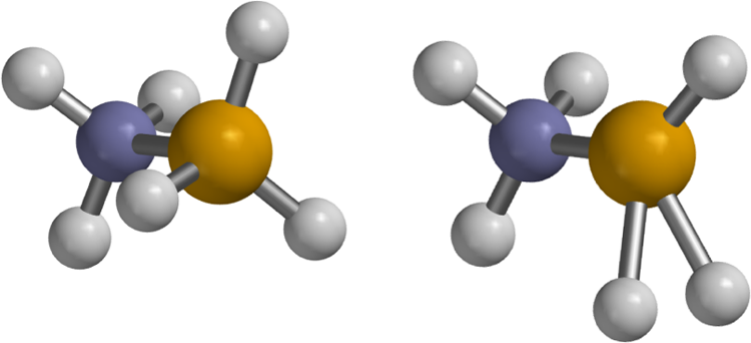
Ground state geometry of the AB neutral (left) and dication (right), visualized using Spartan ’24.^65^ The nitrogen atom is blue and the boron atom is tan.

Additionally, the hydrogens involved in the elongated bonds of the lowest singlet state of AB^2+^ have an internuclear distance of 0.80 Å comparable to the 0.74 Å bond length of molecular H_2_, thus fulfilling the second condition. The adiabatic relaxation energy of AB^2+^ (7.17 eV) is much higher than the H_2_ dissociation energy (1.23 eV), similar to CH_3_F^2+^ (4.5 and 2.2 eV, respectively).^20^ The larger energy difference in AB^2+^ favors neutral H_2_ release and results in a low H_3_^+^ yield, similar to CH_3_F^2+^.^64^ Nevertheless, the observation of H_3_^+^ from AB indicates that the same governing factors extend beyond methyl pseudohalogens and halogens, enabling a framework for predicting when H_2_ release will lead to appreciable H_3_^+^ formation and providing additional insight into the mechanisms of H_2_ release and subsequent H_3_^+^ production in AB.

We performed ab initio molecular dynamics (MD) simulations for both the singly and doubly charged cations of BH_3_NH_3_, using 300 independent trajectories conserving the number of particles, volume, and energy. In all cases, the initial atomic positions and velocities were sampled from a 300 K MD simulation of the neutral parent molecule, so the maximum energy available for fragmentation corresponds to the adiabatic relaxation energy following ionization.

Under these conditions, the singly charged cation rarely undergoes hydrogen loss, likely due to the 0.74 eV H-bond dissociation energy.^54^ AIMD trajectories of the monocation were performed with total nuclear kinetic energies of 0, 1, 1.5, and 7.8 eV. 7.8 eV was chosen because the energy difference between the 1e_1_ state and the ground state of the AB cation is 7.76 eV. The 1 and 1.5 eV trajectories were extended to 2 ps in order to capture fragmentation pathways occurring on longer time scales. The results of monocation AIMD trajectories are summarized in Figure 6. As the total kinetic energy increases, the yield of intact AB decreases while H and/or H_2_ loss becomes more prominent. This dissociation originates exclusively from the boron end of the molecule up to 7.8 eV of deposited energy, consistent with the lower bond dissociation energy of B–H relative to N–H bonds. The increased yield of *m*/*q* 30, corresponding to the loss of one H, with increasing internal energy is consistent with the high experimental yield observed for *m*/*q* 30.

**Figure 6:**
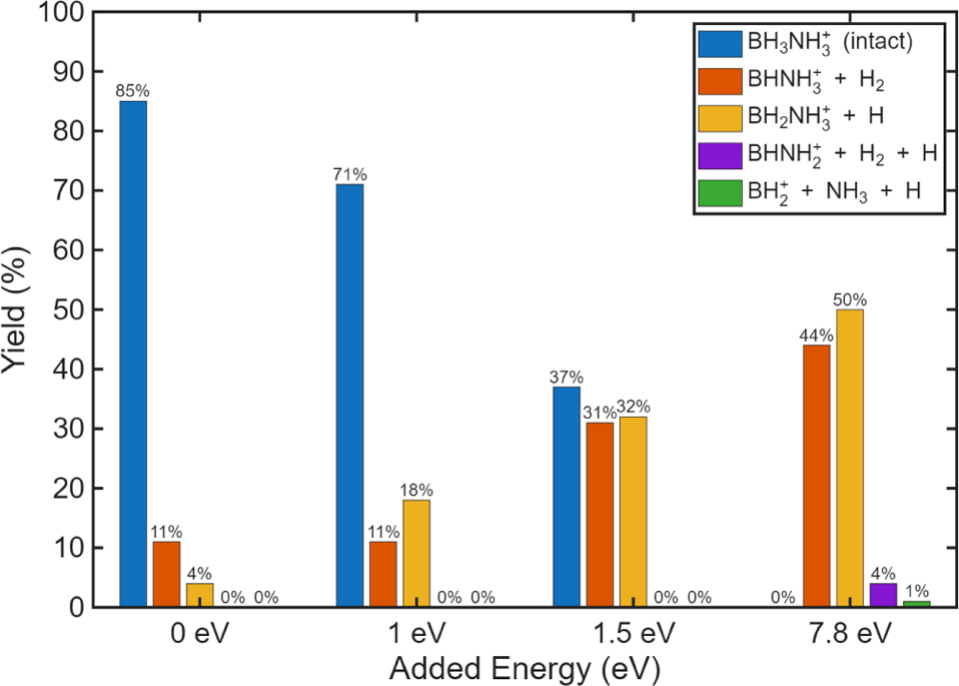
AIMD trajectory results for the monocation for 0, 1, 1.5, and 7.8 eV kinetic energy.

Furthermore, we performed AIMD trajectories for the dication (see Movie 1 for a H_3_^+^ formation trajectory, Movie 2 for a B–N bond cleavage trajectory, Movie 3 for a H_3_^+^ formation trajectory following H scrambling, and Figure S5 for snapshots of these trajectories). After 1 ps, 95% of the BH_3_NH_3_^2+^ trajectories exhibit hydrogen loss, with product yields following the trend

$$H+H^{+}>{H_{2}}^{+}>H_{2}>H^{+}>{H_{3}}^{+}$$

­(see Table 4). Following hydrogen loss, BHNH_3_^2+^ is the most relevant dication for subsequent fragmentation.

**Table 4:** Primary Hydrogen-Release Channels from BH_3_NH_3_^2+^ Observed from Ab Initio Molecular Dynamics (MD) Simulations^a^

channel	yield [%]	time [fs]
${BH}_{3}{{NH}_{3}}^{2+}\to intact$	5	-
${BH}_{3}{{NH}_{3}}^{2+}\to {{BHNH}_{3}}^{+}+H+H^{+}$	33	73 ± 55
${BH}_{3}{{NH}_{3}}^{2+}\to {{BHNH}_{3}}^{+}+{H_{2}}^{+}$	23	134 ± 74
${BH}_{3}{{NH}_{3}}^{2+}\to {{BHNH}_{3}}^{2+}+H_{2}$	19	194 ± 120
${BH}_{3}{{NH}_{3}}^{2+}\to {BH}_{2}{{NH}_{3}}^{+}+H^{+}$	15	47 ± 23
${BH}_{3}{{NH}_{3}}^{2+}\to {{BNH}_{3}}^{+}+{H_{3}}^{+}$	0.5	-
${BH}_{3}{{NH}_{3}}^{2+}\to {{BHNH}_{2}}^{+}+{H_{3}}^{+}$	3.5	299 ± 127

a Initial atomic positions and velocities were sampled from a 300 K MD trajectory of neutral BH_3_NH_3_. After ionization, trajectories of the dication were propagated in the *NVE* ensemble.

The average kinetic energy of nascent BHNH_3_^2+^ is 1.7 eV, which is insufficient to cleave the B–N bond (dissociation energy ∼ 3.7 eV, see Figure S5). However, this barrier can be overcome experimentally through laser-induced electron recollision. At the laser intensities used here, the recollision energy can exceed 50 eV, which rapidly redistributes as electronic and vibrational excitation. To simulate such conditions and explore B–N bond dissociation, we performed additional MD trajectories for vibrationally excited dications. In these simulations, vibrational energy was injected by either: (i) distributing 5 or 7 eV of kinetic energy among all atoms according to a Boltzmann distribution, or (ii) selectively allocating 4 eV into the normal mode with the largest B–N stretch character (using quasi-classical sampling^66^).

The resulting fragmentation pathways are summarized in Figure 7, which shows that H loss in the form of H^+^, H, H_2_^+^, and H_2_ are predominantly released from the boron atom. This observation is consistent with the literature, which shows that BH_2_NH_3_^+^ is the most abundant ionization product of (NH_3_BH_3_)_*n*_^+^ (*n* = 1–3). Its formation involves a small energy barrier of 0.67 eV, indicating preferential hydrogen loss from the boron center.^67^ In addition, studies of the dissociative photoionization of ammonia borane have likewise demonstrated hydrogen loss occurring at the boron center.^54^ Even when 4 eV are injected into the vibrational mode that stretches the B–N bond, H_2_ is formed approximately 60% of the time. The molecular dynamics simulations support the following assignments for the strong-field ionization mass spectrum: (i) most abundant dication, (M – 2H)^2+^, corresponds to BHNH_3_^2+^. (ii) Most abundant cation, (M – 3H)^+^, corresponds to BHNH_2_^+^. (iii) NH_3_^+^ and NH_2_^+^ can be formed from Coulomb explosion through two channels, while NH^+^ is only significantly observed from fragmentation of BNH_2_^2+^. This explains why NH_3_^+^ is as abundant as NH_2_^+^ and more than NH^+^.

**Figure 7:**
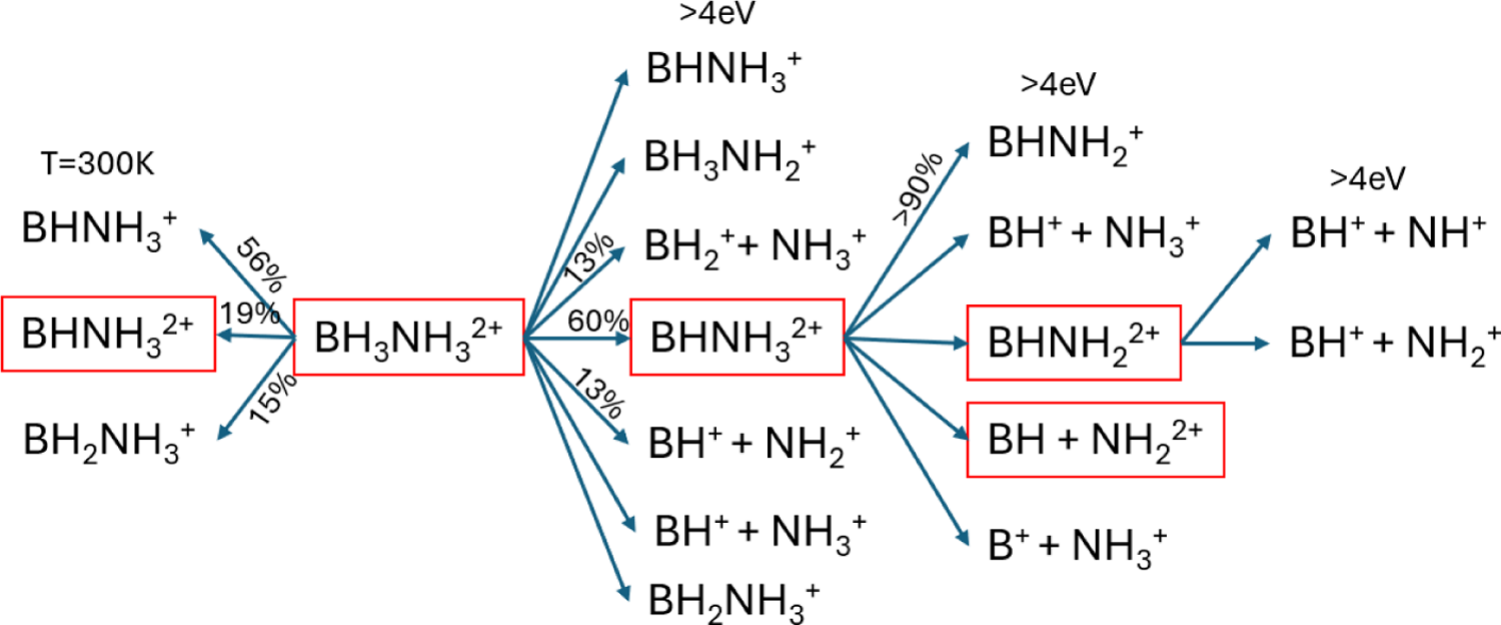
Fragmentation pathways of the doubly charged cation BH_3_NH_3_^2+^ (red rectangles) following sequential dissociation events. Values accompanying arrows indicate the fraction of trajectories resulting in each specific fragment or set of fragments. Released H’s are not indicated for clarity. Numbers on top of each column denote the minimum vibrational energy (in eV) injected into the parent cation during the initial momentum assignment. The leftmost column corresponds to a purely thermal sampling at 300 K, with no additional vibrational energy. Only the most abundant products and primary pathways are shown.

In general, we never observe a trajectory resulting in BH_3_^+^, which suggests that the peak in the mass spectrum at *m*/*q* = 14 primarily corresponds to N^+^. Works on the catalytic dehydrogenation of neutral AB show that it is easier to abstract H from B than N,^68^ consistent with our findings. Summarizing, MD suggests that upon double ionization, BH_3_NH_3_^2+^ undergoes H atom/H_2_-molecule elimination prior to Coulomb explosion yielding BH^+^ > BH_3_^+^, and NH_3_^+^ ∼ NH_2_^+^ > NH^+^ fragments. Also, under single ionization, atomic and molecular hydrogen are most likely released as neutral species.

## Conclusions

In conclusion, we performed time-resolved strong-field ionization of ammonia borane and analyzed its ultrafast fragmentation dynamics. Hydrogen release was observed from both monocationic and dicationic species and occurs on subpicosecond time scales. Through disruptive probing, we identified that the loss of two and four hydrogen atoms, corresponding to *m*/*q* 29 and 27, originates from a common fragmentation pathway and proceeds sequentially. The released hydrogen species predominantly originate from the boron site. Notably, the monocation produces only neutral H and H_2_. These data also enable the determination of their respective kinetic energy release distributions.

High-level electronic structure calculations were used to determine the first and second ionization potentials of AB, as well as the adiabatic relaxation energy and geometry of the lowest singlet state of the dication. These confirm the AB dication satisfies the structural and energetic criteria for H_2_ release, a prerequisite for forming H_3_^+^. However, the high adiabatic energy causes most H_2_ molecules to leave instead of roaming and abstracting a proton. These results extend principles established for the formation of H_3_^+^ via the H_2_ roaming mechanism for methyl halogens and pseudohalogens to AB, improving our understanding of far-from-equilibrium chemical processes under strong-field conditions.

AIMD trajectories of the dication reveal extensive H scrambling and H_3_^+^ formation via a neutral roaming H_2_ intermediate, while also showing that B–N bond cleavage requires substantially higher energy; consistent with experiment, as the earliest H loss is initiated from the borane end of the molecule. Given the high hydrogen content of AB and its importance as a promising chemical hydrogen storage material, understanding its gas-phase ionization and fragmentation dynamics provides valuable insight into its dissociative behavior and potential applications.

## Supplementary Material



## Notes

It contains: (1) details about how the threshold laser intensity proportional to the appearance energies were estimated (S-1); (2) comparison of high and low intensity dynamics for *m*/*q* 29 (S-3); (3) average of 39,660 mass spectra of AB used for calculating the kinetic energy release (S-4); (4) AIMD movie snapshots for three trajectories (S-6); (5) cuts along the potential energy surface as a function of B–N bond distance (S-7); and (6) table with primary hydrogen-release channels from AB (S-8) (PDF)
