# Supplementary material for: Ultrafast Cation–Dication Dynamics in Ammonia Borane: H‑Migration to Roaming H2 and Reduced H3 + Formation under Strong-Field Ionization
Source: J Phys Chem A. 2026 Feb 20;130(9):1780–9. doi: 10.1021/acs.jpca.5c07228 (PMC12969360; doi:10.1021/acs.jpca.5c07228)
Supplement: Supplementary file 1 [file jp5c07228_si_001.pdf]

Supporting Information: Ultrafast cation-dication dynamics in ammonia borane: H-migration to roaming H_2 and reduced H_3^+ formation under strong-field ionization

Sung Kwon,[†] Naga Krishnakanth Katturi,[†] Bruno I. Moreno,[‡] Carlos Cárdenas,[*,‡] and Marcos Dantus[*,†,¶,§]

†*Department of Chemistry, Michigan State University, 48824 East Lansing, MI, United States*

‡*Department of Physics, University of Chile, CEDENNA, Las Palmeras, 3425, Ñuñoa, Chile*

¶*Department of Physics and Astronomy, Michigan State University, 48824 East Lansing, MI, United States*

§*Department of Electric and Computer Engineering, Michigan State University, 48824 East Lansing, MI, United States*

E-mail: cardena@uchile.cl; dantus@chemistry.msu.edu

Table of Contents

1 Appearance Energy Estimation **S-3**

2 Comparison of High and Low Intensity Dynamics **S-4**

3 Mass Spectrum of AB for Correlation Coefficient Analysis **S-5**

4 AIMD Movie Snapshots **S-6**

5 B-N Bond Breaking Energy **S-7**

6 Primary hydrogen-release channels from $BH_3NH_3^+$ **S-8**

1 Appearance Energy Estimation

Included here is an illustration of the procedure used to extract appearance intensities from the intensity-dependent fragment yields. In our experiments, ionization occurs in the tunneling regime, and the electron recollision probability scales with the laser intensity. Therefore, to a good approximation, we take the appearance intensity to be proportional to the appearance energy. For each fragment, the integrated yield is converted to a difference trace by subtracting point n from point n+1, which suppresses focal-volume contributions. The resulting trace is fit with an error function, and the onset intensity is obtained from the fitted curve (arrow shown for m/q 18). This same workflow is applied to the major fragments summarized in the intensity-dependent heat map in Figure 2 of the main text.

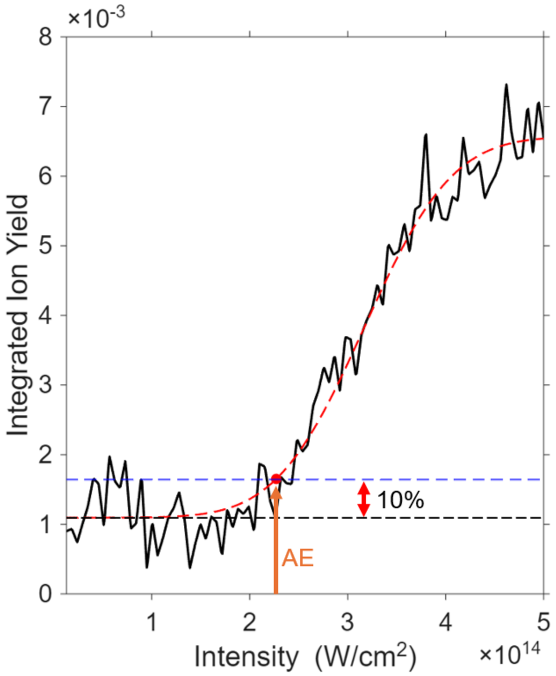

Figure S1: The integrated ion yield of m/q 18 as a function of laser intensity. The black line corresponds to the experimental data while the red dashed line is the error function fit. The orange arrow indicates where the appearance intensity of m/q 18 is estimated.

2 Comparison of High and Low Intensity Dynamics

The following figure is included to demonstrate that pump–probe transients recorded at nominally high intensity can be dominated by contributions from lower-intensity regions of the interaction volume when no intensity-selection procedure is applied. We test this explicitly using the m/q 29 channel (loss of two H atoms): the delay-dependent ion yield measured at 3.1×10^{14} W/cm^2 closely matches the transient measured at 1.1×10^{14} W/cm^2. The near overlap of these traces supports the conclusion that the apparent high-intensity dynamics are strongly weighted by lower-intensity contributions, motivating the high-intensity selection approach used in the main analysis.

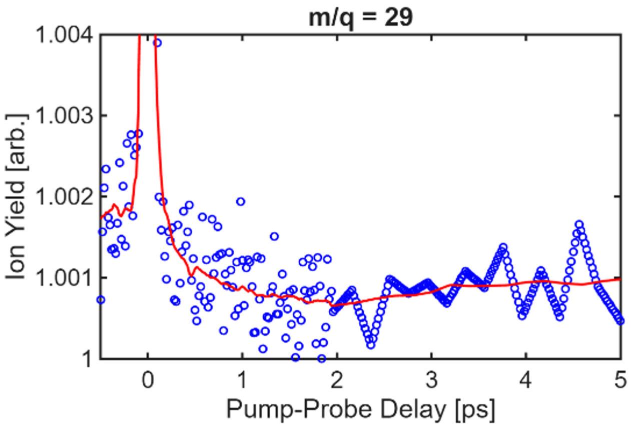

Figure S2: Comparison of ion yields for m/q 29 as a function of pump–probe delay at high intensity (red line, 3.1×10^{14} W/cm^2) and low intensity (blue circles, 1.1×10^{14} W/cm^2).

3 Mass Spectrum of AB for Correlation Coefficient Analysis

Here we highlight the quality and key features of the mass spectrum used for the kinetic energy analyses. The figure shows a representative portion of the average of 39,660 single-shot mass spectra acquired at 3.1×10^{14} W/cm^2, highlighting that many peaks in the m/q 2–17 region exhibit distinct forward and backward components characteristic of Coulomb explosion. These split features provide the basis for the KER analysis (Figure 5) in the main text.

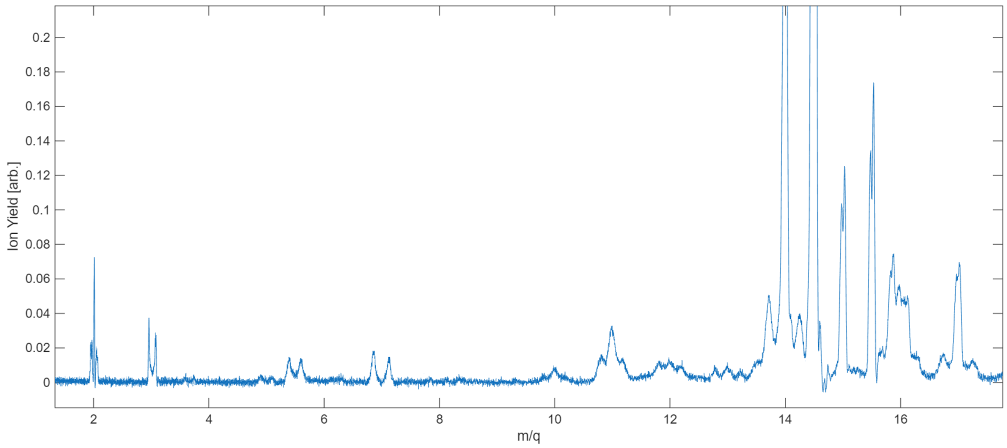

Figure S3: Zoomed in MS of AB from m/q 2 to 17.

4 AIMD Movie Snapshots

To complement the experimental fragment-correlation analysis, we performed ab initio molecular dynamics (AIMD) simulations on doubly ionized ammonia borane AB_2^+ to directly visualize the ultrafast structural rearrangements and dissociation pathways accessible after double ionization. Representative trajectory snapshots are presented below, highlighting H_3^+ formation, B–N bond cleavage, and H_3^+ formation following hydrogen scrambling (see Movies 1–3).

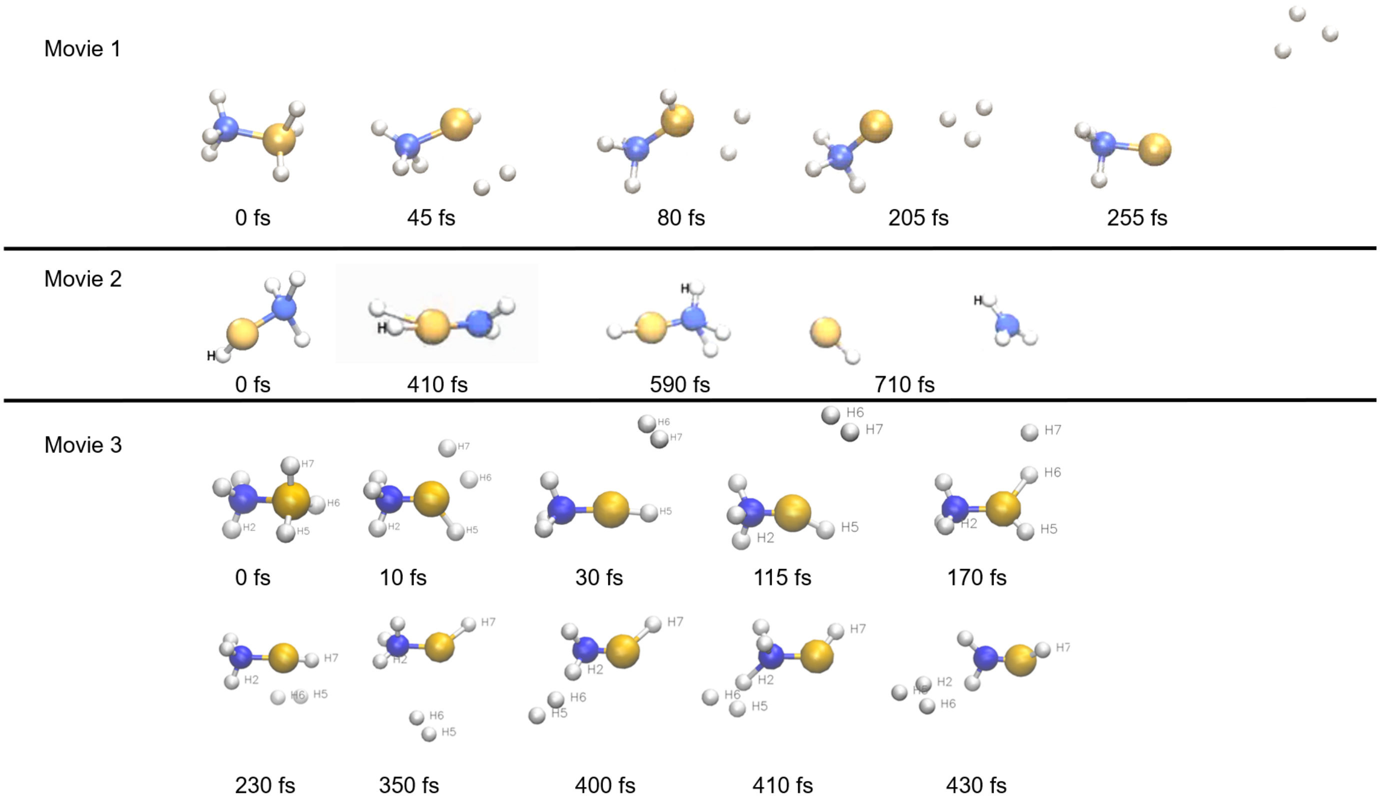

Figure S4: The ab initio molecular dynamics trajectory snapshots show the formation of H_3^+ from doubly charged ammonia borane (AB), B–N bond cleavage following double ionization, and H_3^+ formation from doubly charged AB after hydrogen scrambling.

5 B-N Bond Breaking Energy

To help interpret the bond-cleavage energetics, we include one-dimensional cuts of the potential energy surface along the B–N stretch coordinate. The plot reports the electronic energy as a function of the B–N distance, with each curve referenced to its own ground-state minimum (zero energy). For $BH_3NH_3^+$, the curve exhibits a discontinuity near 2.9 Å that reflects a change in the dominant reaction coordinate: as the B–N bond is elongated, one hydrogen migrates from boron to nitrogen (not shown), leading to formation of the separated products BH_2 and NH_4^+ rather than simple homolytic B–N bond dissociation.

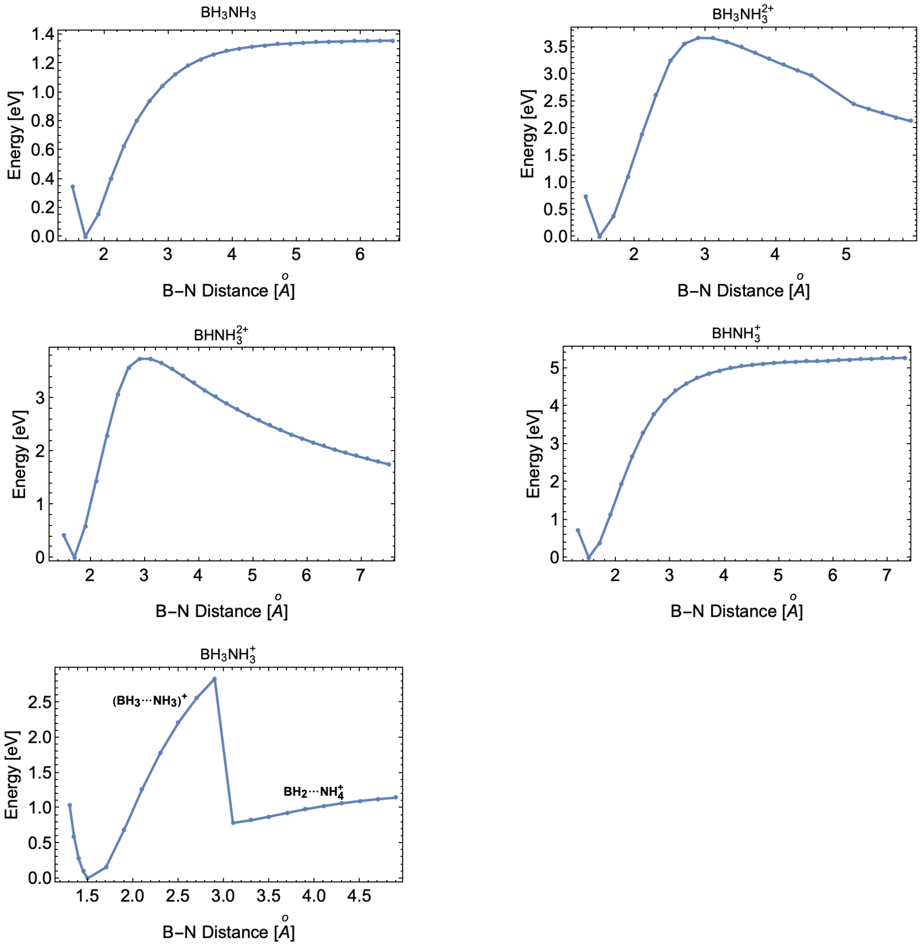

Figure S5: Potential energy as a function of the B-N distance. The zero energy is set to the ground state energy of each compound. In the case of $BH_3NH_3^+$, the discontinuity around 2.9 Å corresponds to the migration of one of the H from B to N (not shown) to form the products BH_2 and NH_4^+.

6 Primary hydrogen-release channels from $BH_3NH_3^+$

Table S1: Primary hydrogen-release channels from $BH_3NH_3^+$ observed from ab initio molecular dynamics (MD) simulations. Initial atomic positions were sampled from a 300 K MD trajectory of neutral BH_3NH_3. After ionization, trajectories of the cation were propagated in the NVE ensemble. Velocities in the NVE ensemble were thermally sampled ($BH_3NH_3^+$) or adjusted to distribute 1 and 1.5 eV of kinetic energy among all atoms according to a Boltzmann distribution ($BH_3NH_3^+$ @ 1 eV or @ 1.5 eV)

**Channels $BH_3NH_3^+$**	**Yield**
$BH_3NH_3^+ \rightarrow$ intact	85%
$BH_3NH_3^+ \longrightarrow BHNH_3^+ + H$	4%
$BH_3NH_3^+ \longrightarrow BHNH_3^+ + H_2$	11%
**Channels $BH_3NH_3^+$ @ 1 eV**	**Yield**
$BH_3NH_3^+ \rightarrow$ intact	71%
$BH_3NH_3^+ \longrightarrow BHNH_3^+ + H$	18%
$BH_3NH_3^+ \longrightarrow BHNH_3^+ + H_2$	11%
**Channels $BH_3NH_3^+$ @ 1.5 eV**	**Yield**
$BH_3NH_3^+ \rightarrow$ intact	37%
$BH_3NH_3^+ \longrightarrow BHNH_3^+ + H$	32%
$BH_3NH_3^+ \longrightarrow BHNH_3^+ + H_2$	31%
